# Association between occupation type and development of type 2 diabetes: A population-based Panasonic cohort study 3

**DOI:** 10.3389/fpubh.2023.1103275

**Published:** 2023-01-19

**Authors:** Momoko Habu, Hiroshi Okada, Masahide Hamaguchi, Kazushiro Kurogi, Hiroaki Murata, Masato Ito, Michiaki Fukui

**Affiliations:** ^1^Department of Diabetes and Endocrinology, Matsushita Memorial Hospital, Moriguchi, Japan; ^2^Department of Endocrinology and Metabolism, Graduate School of Medical Science, Kyoto Prefectural University of Medicine, Kyoto, Japan; ^3^Department of Health Care Center, Panasonic Health Insurance Organization, Moriguchi, Japan; ^4^Department of Orthopaedic Surgery, Matsushita Memorial Hospital, Moriguchi, Japan

**Keywords:** type 2 diabetes, occupation type, Japanese, risk factors, cohort study

## Abstract

**Background:**

Due to a lack of investigation on the association between the type of occupation and the development of type 2 diabetes among Japanese individuals, we aimed to assess this association in 98,935 Japanese individuals.

**Methods:**

This long-term retrospective cohort study included participants selected from medical health checkup programs conducted at the Panasonic Corporation, Osaka, Japan, from 2008 to 2018. Cox regression analyses were used to evaluate the association between occupation type and the incidence of type 2 diabetes.

**Results:**

From 2008 to 2018, 5,008 participants developed type 2 diabetes. The proportion of never smokers, those with slow eating speeds, and those working with a flextime system was higher in men with technical jobs than in salespersons, manufacturers, and office workers (*p* < 0.0001). Cox regression analyses revealed that occupation type was associated with an increased probability of type 2 diabetes development in men but not in women. Multivariate analyses showed that the hazard ratios were 1.15 [95% confidence interval (CI), 1.05–1.26], 1.20 (95% CI, 1.10–1.30), and 1.11 (95% CI, 1.02–1.21) in men working as salespersons, manufacturers, and office workers, respectively (reference group: men with technical jobs). On the other hand, the occupation type was not associated with the development of type 2 diabetes in women.

**Conclusions:**

This study demonstrated that occupation type might be an independent factor in the development of type 2 diabetes in Japanese men.

## 1. Introduction

The global prevalence of diabetes and its associated medical costs have increased tremendously over the past decade. Therefore, it is essential to prevent the development and progression of diabetes in clinical settings.

Work-related factors, including working hours, socioeconomic status, and job insecurity, have reportedly been associated with cardiovascular disease, mortality, and diabetes development (1–6). As work-related factors are associated with unhealthy lifestyle habits, stress, sleep disturbances, and symptoms of depression, they contribute to the development of diabetes (7, 8). Moreover, Kivimäki et al. (5) reported in their meta-analysis that the correlation between working hours and the development of type 2 diabetes varies by occupation type. Moreover, occupation type is also considered as one of the major work-related factors. However, few studies have investigated the association between occupation type and the development of diabetes in the Japanese population. Although Osaki et al. (9) reported an association between shift work and the development of type 2 diabetes, they did not consider the occupation of the participants. Nagaya et al. (10) suggested that salespersons had a higher risk of type 2 diabetes than those in other occupations. However, their study included only men and did not include analysis of plasma glucose levels in their multivariate analyses, which is one of the most important factors for the development of type 2 diabetes.

Thus, it is unclear whether occupation type is associated with the development of type 2 diabetes in the Japanese population. Therefore, the aim of the present study was to investigate the association between occupation types and the development of type 2 diabetes among Japanese populations in a large cohort.

## 2. Materials and methods

### 2.1. Study design and data collection

This long-term retrospective cohort study included participants selected from medical health checkup programs conducted at the Panasonic Corporation, Osaka, Japan. All employees participated in this program every year. We used the data collected between 2008 and 2018 from the Panasonic cohort study database. This particular Panasonic cohort study has been described in detail elsewhere (11). We used a questionnaire, which was previously standardized and validated self-administered, to evaluate baseline characteristics. Participants were classified as non-smokers, current smokers, or past smokers. The participants were also categorized into three levels of eating speed: fast, normal, and slow. We also asked the participants of their breakfast habits and habits of snack after dinner. No alcohol consumption was defined as daily alcohol consumption <20 g/day for women and <30 g/day for men. Regular exercisers were defined as participants who regularly exercised for at least 30 min at least 2 days per week for at least 1 year. We also asked the sleeping hours of participants. Here, the participants were questioned about their working style and the details regarding flextime and night shifts. Occupation types present in this study included technical jobs, salespersons, manufacturers, and office workers. The participants were classified in a particular category based on the following criteria: the people who had specific talent/ expertise or engaged in a product development job were classified as those with technical jobs. A salesperson was defined as the person who persuaded people to buy the company's products; manufacturers included individuals who made products for their company, and office workers were those who engaged in paperwork or office work. Type 2 diabetics was defined as having a fasting plasma glucose level of ≥126 mg/dL or, a self-reported history of diabetes, and/or the use of anti-diabetic medication.

### 2.2. Ethics approval

This study was approved by the local ethics committee of the Panasonic Health Insurance Organization (approval number: 2021-001) and conducted in accordance with the principles of the Declaration of Helsinki.

### 2.3. Exclusion criteria

In total, 236,603 employees underwent medical health checkups between 2008 and 2018. Participants who did not undergo a blood examination at baseline (n = 74,827), those with missing data or unknown occupation type (*n* = 27,464), those with diabetes at baseline (*n* = 7,096), and those who had undergone a medical health checkup only at baseline (*n* = 28,281) were excluded from the study.

### 2.4. Statistical analyses

The mean and frequency of the potential confounding variables were calculated. The participants were classified according to sex. The general characteristic differences at baseline were categorized according to occupation type or diabetes development during follow-up and were assessed using analysis of variance, the *t*-test, or the chi-square test as appropriate. Group comparisons were conducted using the Tukey–Kramer (continuous variables) or the Bonferroni method (categorical variables). The association between the occupation type and the development of type 2 diabetes was evaluated using Cox regression analyses and multivariate models. The covariates included in the multivariate model are known to be associated with the development of type 2 diabetes. Additionally, the multivariate model (Model 1) was adjusted for age, body mass index (BMI), systolic blood pressure (SBP), low-density lipoprotein (LDL) cholesterol, high-density lipoprotein (HDL) cholesterol, triglyceride, and glucose levels. Model 2 included factors of as model 1 as well as details on smoking status, eating speed, breakfast skipping, snacking after dinner, alcohol consumption, physical exercise, flextime system, night shifts, and hours of sleep. All continuous variables are presented as mean ± SD or absolute number. *P* < 0.05 were considered statistically significant. While using the Bonferroni method, differences were considered statistically significant at *p* < 0.008. The associations were presented as hazard ratios (HR) with 95% confidence intervals. All statistical analyses were performed using JMP software version 10 (SAS Institute, Cary, NC, USA).

## 3. Results

The baseline characteristics of the participants enrolled in this study are presented in Tables 1, 2. Men with technical jobs were younger than the salespersons, manufacturers, and office workers included in this study. SBP was lower in men with technical jobs than in those working as salespersons, manufacturers, and office workers. The proportion of never smokers, those with slow eating speeds, and those working with a flextime system was higher in men with technical jobs than the others. Further, the proportion of alcohol drinkers and those with exercise habits was lower among men with technical jobs than among those working as salespersons, manufacturers, and office workers. In addition, the proportion of never-smokers and those working with a flextime system was higher in women with technical jobs than in those working as salespersons, manufacturers, and office workers.

**Table 1:** Characteristics of patients at baseline according to occupation type in men.

	**All**	**Technical jobs**	**Salesperson**	**Manufacturer**	**Office worker**	***p***
*n*	76,092	27,038	12,303	20,965	15,786	
Age (y)	45.0 (8.2)	44.0 (7.8)	44.4 (8.9)^*^	44.5 (8.3)^*^	47.9 (7.6)^*^	< 0.0001
Body mass index (kg/m^2^)	23.4 (3.2)	23.2 (3.1)	24.1 (3.2)^*^	23.1 (3.3)^*^	23.7 (3.1)^*^	< 0.0001
Systolic blood pressure (mmHg)	120.5 (14.0)	119.3 (13.6)	121.7 (14.4)^*^	120.8 (14.0)^*^	121.0 (14.2)^*^	< 0.0001
Diastolic blood pressure (mmHg)	75.6 (10.6)	75.0 (10.4)	76.4 (11.0)^*^	75.0 (10.5)	76.7 (10.5)^*^	< 0.0001
LDL cholesterol (mg/dL)	126.0 (31.2)	126.0 (30.7)	125.7 (31.6)	124.8 (31.9)^*^	127.9 (30.6)^*^	< 0.0001
HDL cholesterol (mg/dL)	57.7 (14.2)	57.5 (13.9)	57.4 (14.2)	58.3 (14.6)^*^	57.4 (14.2)	< 0.0001
Triglyceride (mg/dL)	122.0 (93.1)	116.2 (84.3)	136.4 (108.3)^*^	118.0 (91.0)	126.1 (95.8)^*^	< 0.0001
Glucose (mg/dl)	93.9 (9.3)	93.8 (8.9)	93.5 (9.5)^*^	93.6 (9.7)	94.8 (9.2)^*^	< 0.0001
Smoking (none/past/current)	32,964/13,527/29,601 (43.3/17.8/38.9)	14,444/4,859/7,735 (53.4/18.0/28.6)	4,610/2,363/5,330^*^ (37.5/19.2/43.3)	6,914/2,996/11,055^*^ (33.0/14.3/52.7)	6,996/3,609/5,481^*^ (44.3/21.0/34.7)	< 0.0001
Eating speed (fast/normal/slow)	26,847/44,212/5,033 (35.3/58.1/6.6)	9,240/15,760/2,038 (34.2/58.3/7.5)	5,133/6,480/690^*^ (41.7/52.7/5.6)	6,651/12,984/1,330^*^ (31.7/61.9/6.3)	5,823/8,988/975^*^ (36.9/56.9/6.2)	< 0.0001
Skipping breakfast (+/–)	16,983/59,109 (22.3/77.7)	5,031/22,007 (18.6/81.4)	3,679/8,624^*^ (29.9/70.1)	5,627/15,338^*^ (26.8/73.2)	2,646/13,140^*^ (16.8/83.2)	< 0.0001
Snack after dinner (+/–)	12,707/63,385 (16.7/83.3)	4,442/22,596 (16.4/83.6)	1,750/10,553^*^ (14.2/85.8)	4,230/16,735^*^ (20.2/79.8)	2,285/13,501^*^ (14.5/85.5)	< 0.0001
Alcohol drinker (+/–)	8,581/67,511 (11.3/88.7)	2,104/24,934 (7.8/92.2)	2,190/10,113^*^ (17.8/82.2)	2,382/18,583^*^ (11.4/88.6)	1,905/13,881^*^ (12.1/87.9)	< 0.0001
Physical exercise (+/–)	14,242/61,850 (18.7/81.3)	4,666/22,372 (17.3/82.7)	2,399/9,904^*^ (19.5/80.5)	3,981/16,984^*^ (19.0/81.0)	3,196/12,590^*^ (20.2/79.8)	< 0.0001
Flextime system (+/–)	24,927/51,165 (32.8/67.2)	15,201/11,837 (56.2/43.8)	2,819/9,484^*^ (22.9/77.1)	1,803/19,162^*^ (8.6/91.4)	5,104/10,682^*^ (32.3/67.7)	< 0.0001
Night shift (+/–)	15,468/83,467 (15.6/84.4)	2,477/24,561 (9.2/90.8)	1,478/10,825^*^ (12.0/88.0)	9,206/11,759^*^ (43.9/56.1)	1,179/14,607^*^ (7.5/92.5)	< 0.0001
Hours of sleep; ≤ 5 h (+/–)	16,210/59,882 (21.3/78.7)	6,157/20,881 (22.8/77.2)	2,829/9,474 (23.0/77.0)	4,165/16,800^*^ (19.9/80.1)	3,059/12,727^*^ (19.4/80.6)	< 0.0001

Data are expressed as mean (SD) or absolute number. LDL, Low-density lipoprotein; HDL, High-density lipoprotein.

* Significant difference compared to technical jobs.

**Table 2:** Characteristics of patients at baseline according to occupation type in women.

	**All**	**Technical jobs**	**Salesperson**	**Manufacturer**	**Office worker**	***p***
*n*	22,843	2,144	1,491	5,948	13,260	–
Age (y)	41.2 (8.2)	40.4 (7.9)	36.6 (9.8)^*^	44.4 (7.8)^*^	40.4 (7.8)	< 0.0001
Body mass index (kg/m^2^)	21.4 (3.5)	21.2 (3.4)	20.9 (3.0)	22.2 (3.8)^*^	21.1 (3.4)	< 0.0001
Systolic blood pressure (mmHg)	112.0 (14.3)	110.5 (13.6)	109.1 (13.6)^*^	115.6 (14.9)^*^	110.9 (14.0)	< 0.0001
Diastolic blood pressure (mmHg)	68.4 (10.4)	67.8 (10.3)	66.5 (9.9)^*^	70.1 (10.5)^*^	68.0 (10.4)	< 0.0001
LDL cholesterol (mg/dL)	114.3 (30.5)	111.7 (29.8)	107.9 (29.3)^*^	120.1 (31.8)^*^	112.8 (29.8)	< 0.0001
HDL cholesterol (mg/dL)	70.0 (14.9)	69.7 (14.6)	70.7 (15.0)	68.6 (14.9)^*^	70.5 (14.9)	< 0.0001
Triglyceride (mg/dL)	71.3 (44.6)	71.4 (47.0)	68.2 (39.7)	75.7 (49.2)^*^	69.7 (42.3)	< 0.0001
Glucose (mg/dl)	88.1 (8.2)	88.3 (7.6)	86.2 (7.7)^*^	89.7 (8.7)^*^	87.5 (8.0)^*^	< 0.0001
Smoking (none/past/current)	18,135/1,660/3,048 (79.4/7.3/13.3)	1,838/127/179 (85.7/5.9/8.3)	1,123/139/229^*^ (75.3/9.3/15.4)	4,534/320/1,094^*^ (76.2/5.4/18.4)	10,640/1,074/1,546^*^ (80.2/8.1/11.7)	< 0.0001
Eating speed (fast/normal/slow)	5,822/14,636/2,385 (25.5/64.1/10.4)	530/1,356/258 (24.7/63.2/12.0)	469/846/176^*^ (31.5/56.7/11.8)	1,353/4,108/487 (22.7/69.1/8.2)	3,470/8,326/1,464^*^ (26.2/62.8/11.0)	< 0.0001
Skipping breakfast (+/–)	4,002/18,841 (17.5/82.5)	370/1,774 (17.3/82.7)	424/1,067^*^ (28.4/71.6)	885/5,063 (14.9/85.1)	2,323/10,937 (17.5/82.5)	< 0.0001
Snack after dinner (+/–)	5,377/17,466 (23.5/76.5)	445/1,699 (20.8/79.2)	315/1,176 (21.1/78.9)	1,555/4,393 (26.1/73.9)	3,062/10,198^*^ (23.1/76.9)	< 0.0001
Alcohol drinker (+/–)	2,006/20,837 (8.8/91.2)	194/1,950 (9.0/91.0)	169/1,322 (11.3/88.7)	393/5,555 (6.6/93.4)	1,250/12,010^*^ (9.4/90.6)	< 0.0001
Physical exercise (+/–)	2,599/20,244 (11.4/88.6)	250/1,894 (11.7/88.3)	180/1,311 (12.1/87.9)	596/5,352 (10.0/90.0)	1,573/11,687 (11.9/88.1)	0.002
Flextime system (+/–)	3,628/19,215 (15.9/84.1)	887/1,257 (41.4/58.6)	295/1,196^*^ (19.8/80.2)	253/5,695^*^ (4.3/95.7)	2,193/11,067^*^ (16.5/83.5)	< 0.0001
Night shift (+/–)	1,128/21,715 (4.9/95.1)	118/2,026 (5.5/94.5)	86/1,405 (5.8/94.2)	665/5,283^*^ (11.2/88.8)	259/13,001^*^ (2.0/98.0)	< 0.0001
Hours of sleep; ≤ 5 h (+/–)	5,050/17,793 (22.1/77.9)	533/1,611 (24.9/75.1)	343/1,148 (23.0/77.0)	1,341/4,607^*^ (22.5/77.5)	2,833/10,427 (21.4/78.6)	0.002

Data are expressed as mean (SD) or absolute number. LDL, Low-density lipoprotein; HDL, High-density lipoprotein.

* Significant difference compared to technical jobs.

Table 3 shows the baseline characteristics of the participants according to the development of type 2 diabetes. In total, 4,642 men and 366 women developed type 2 diabetes between 2008 and 2018. The incidence rate for type 2 diabetes was 1,394 (5.2%), 852 (6.9%), 1,348 (6.4%), and 1,048 (6.6%) in men with technical jobs, salespersons, manufacturers, and office workers, respectively. The incidence rate for type 2 diabetes was 31 (1.5%), 17 (1.1%), 158 (2.7%), and 160 (1.2%) in women with technical jobs, salespersons, manufacturers, and office workers, respectively. We found significant differences between the occupation types according to the development of type 2 diabetes both in men and women.

**Table 3:** Characteristics of participants at baseline according to the development of diabetes.

	**Men**			**Women**		
	**The development of diabetes (–)**	**The development of diabetes (+)**	***p***	**The development of diabetes (–)**	**The development of diabetes (+)**	***p***
*n*	71,450	4,642	–	22,477	366	–
Age (y)	44.9 (8.3)	47.0 (6.5)	< 0.0001	41.1 (8.2)	45.5 (6.4)	< 0.0001
Body mass index (kg/m^2^)	23.3 (3.1)	25.6 (3.9)	< 0.0001	21.3 (3.4)	26.6 (5.8)	< 0.0001
Systolic blood pressure (mmHg)	120.1 (13.8)	126.1 (15.1)	< 0.0001	111.7 (14.1)	126.4 (18.2)	< 0.0001
Diastolic blood pressure (mmHg)	75.3 (10.5)	79.8 (10.8)	< 0.0001	68.3 (10.3)	77.7 (12.3)	< 0.0001
LDL cholesterol (mg/dL)	125.5 (31.0)	133.6 (32.5)	< 0.0001	113.9 (30.4)	137.7 (32.1)	< 0.0001
HDL cholesterol (mg/dL)	58.0 (14.2)	53.1 (13.8)	< 0.0001	70.1 (14.8)	61.1 (14.7)	< 0.0001
Triglyceride (mg/dL)	119.5(90.4)	160.8 (120.7)	< 0.0001	70.7 (44.0)	106.8 (60.5)	< 0.0001
Glucose (mg/dl)	93.1 (8.6)	106.5 (10.8)	< 0.0001	87.8 (7.9)	103.0 (10.8)	< 0.0001
Smoking (none/past/current)	31,303/12,668/27,479 (43.8/17.7/38.5)	1,661/859/2,122 (35.8/18.5/45.7)	< 0.0001	17,849/1,635/2,993 (79.4/7.3/13.3)	286/25/55 (78.1/6.8/15.0)	0.62
Eating speed (fast/normal/slow)	24,825/41,767/4,858 (34.7/58.5/6.8)	2,022/2,445/175 (43.6/52.7/3.8)	< 0.0001	5,693/14,419/2,365 (25.3/64.2/10.5)	129/217/20 (35.3/59.3/5.5)	< 0.0001
Skipping breakfast (+/–)	15,849/55,601 (22.2/77.8)	1,134/3,508 (24.4/75.6)	0.0004	3,930/18,547 (17.5/82.5)	72/294 (19.7/80.3)	0.27
Snack after dinner (+/–)	11,889/59,561 (16.6/83.4)	818/3,824 (17.6/82.4)	0.08	5,289/17,188 (23.5/76.5)	88/278 (24.0/76.0)	0.82
Alcohol drinker (+/–)	7,922/63,528 (11.1/88.9)	659/3,983 (14.2/85.8)	< 0.0001	1,980/20,497 (8.8/91.2)	26/340 (7.1/92.9)	0.25
Physical exercise (+/–)	13,372/58,078 (18.7/81.3)	870/3,772 (18.7/81.3)	0.96	2,559/19,918 (11.4/88.6)	40/326 (10.9/89.1)	0.79
Flextime system (+/–)	23,455/47,995 (32.8/67.2)	1,472/3,170 (31.7/68.3)	0.12	3,550/18,927 (15.8/84.2)	78/288 (21.3/78.7)	0.004
Night shift (+/–)	13,496/57,954 (18.9/81.1)	844/3,798 (18.2/81.8)	0.23	1,108/21,369 (4.9/95.1)	20/346 (5.5/94.5)	0.64
Hours of sleep; ≤ 5 h (+/–)	15,164/56,286 (21.2.78.8)	1,046/3,596 (22.5/77.5)	0.04	4,936/17,541 (22.0/78.0)	114/252 (31.2/68.9)	< 0.0001
Occupation type: technical jobs/salesperson/manufacturer/office workers	25,644/11,451/19,617/14,738 (35.9/16.0/27.5/20.6)	1,394/852/1,348/1,048 (30.0/18.4/29.0/22.6)	< 0.0001	2,113/1,491/5,790/13,100 (9.4/6.6/25.8/58.3)	31/17/158/160 (8.5/4.6/43.2/43.7)	< 0.0001

Data are expressed as mean (SD) or absolute number. LDL, Low-density lipoprotein; HDL, High-density lipoprotein.

Table 4 shows the unadjusted and adjusted HR for the development of type 2 diabetes in men and women, respectively. The type of occupation was associated with an increased probability of developing type 2 diabetes in men. When the reference group was defined as men with technical jobs, the HR was 1.26 [95% confidence interval (CI), 1.16–1.37], 1.38 (95% CI, 1.28–1.49), and 1.17 (95% CI, 1.08–1.27) in salespersons, manufacturers, and office workers, respectively, in model 1. The HR was 1.15 (95% CI, 1.05–1.26), 1.20 (95% CI, 1.10–1.30), and 1.11 (95% CI, 1.02–1.21) in salespersons, manufacturers, and office workers, respectively, in model 2. Interestingly, the occupation type was not associated with the development of type 2 diabetes in women.

**Table 4:** Unadjusted and adjusted hazard ratios (95% CI) for development of diabetes according to occupation type.

	**Men**		**Women**	
**Unadjusted**	**HR**	***P*** **-value**	**HR**	***P*** **-value**
Technical jobs	1	–	1	–
Salesperson	1.48 (1.35–1.61)	< 0.0001	0.87 (0.47–1.56)	0.65
Manufacturer	1.45 (1.35–1.57)	< 0.0001	2.11 (1.46–3.16)	< 0.0001
Office workers	1.48 (1.36–1.60)	< 0.0001	1.04 (0.72–1.56)	0.83
**Adjusted (model 1)**	**HR**	***P*** **-value**	**HR**	***P*** **-value**
Technical jobs	1	–	1	–
Salesperson	1.26 (1.16–1.37)	< 0.0001	1.14 (0.61–2.03)	0.68
Manufacturer	1.38 (1.28–1.49)	< 0.0001	1.44 (0.99–2.17)	0.06
Office workers	1.17 (1.08–1.27)	0.0002	1.10 (0.75–1.65)	0.64
**Adjusted (model 2)**	**HR**	***P*** **-value**	**HR**	***P*** **-value**
Technical jobs	1	–	1	–
Salesperson	1.15 (1.05–1.26)	0.003	1.10 (0.59–1.99)	0.76
Manufacturer	1.20 (1.10–1.30)	< 0.0001	1.41 (0.95–2.17)	0.09
Office workers	1.11 (1.02–1.21)	0.01	1.06 (0.72–1.60)	0.79

Model 1, adjustment for age, body mass index, systolic blood pressure, low-density lipoprotein cholesterol, high-density lipoprotein cholesterol, triglycerides, and fasting plasma glucose.

Model 2, adjusted for model 1 plus smoking status, eating speed, skipping breakfast, snack after dinner, alcohol consumption, exercise habits, flextime system, night shift, and hours of sleep.

## 4. Discussion

To the best of our knowledge, this is the first study to investigate the association between occupation type and the development of type 2 diabetes among the Japanese population in such an extensive cohort. The major finding of this study was that occupation type may be an independent factor for the development of type 2 diabetes in Japanese men but not in women. Our results describe that there is a higher risk of type 2 diabetes development among men who are salespersons, manufacturers, or office workers than among men with technical jobs.

However, the reason for this association remains unclear. It has been reported that there is an increased risk of type 2 diabetes in low-income groups with lower educational qualifications (6, 8). We believe that the educational and income levels of the participants in the present study were almost similar for all occupation types because all participants worked in the same corporation. We thus postulated that working hours, sleeping hours, unhealthy behaviors, and psychosocial stress could affect glucose tolerance. Azami et al. (12) reported an association between long working hours and glycemic control in young Japanese male patients with type 2 diabetes. Such long working hours have been suggested to result in poor glycemic control due to higher job-related stress, which can lead to the development of negative behavioral habits, including overeating and neuroendocrinological problems, causing increased levels of counterregulatory hormones (13). Several reports have suggested that short sleep duration is associated with the development of diabetes (14–16). The results of a meta-analysis suggested a *U*-shaped relationship between sleep duration and risk of type 2 diabetes (14). Moreover, it is well-known that unhealthy behaviors, including smoking, fast eating speed, or skipping breakfast, are associated with the development of diabetes (17–19). We identified similar findings after adjusting sleep duration and unhealthy behaviors in this study. Similarly, variations in psychosocial stress have been reported to be associated with different types of occupation (20). Long-term stress affects the entire neuroendocrine system and can lead to diabetes (21, 22). We believe that occupational stress may differ according to occupation type as the required communication skills, demands of the job, and discretionary powers tend to vary between occupations. Moreover, the proportion of participants working with a flextime system was higher in technical jobs than in those working as salespersons, manufacturers, and office workers. Thus, we postulate that adopting a flextime system may have a positive impact on work stress.

We also assessed why an association between occupation type and the development of type 2 diabetes was observed in men but not in women. A previous study reported sex-based differences in the association between occupation type and job stress. Kawaharada et al. (20) reported that there were associations between job requirements and job stress among occupation types in men but not in women. Thus, these findings support the results of the present study.

The main strengths of the present study were its huge cohort size and the time-span of data collection. However, this study has some limitations. First, it has been reported that working hours, psychosocial stress, exposure to chemicals, seniority in the particular job, educational qualification, salary, and marital/family status were associated with the development of diabetes (1, 5–7, 10, 23–25). Moreover, genetic susceptibility was well-known as the important factor of incident diabetes. Unfortunately, we have no data on these variables. We believe that the educational and income levels of the participants in our study are almost similar for all occupation type because all the participants work in the same corporation. Second, we had no data of quantitative expression of smoking habit and eating speed. Third, the study population comprised only of Japanese men and women with high socioeconomic status; therefore, it is uncertain whether these findings can be generalized to other ethnic groups as well.

## 5. Conclusion

Our study showed that the risk of incident type 2 diabetes was higher in salespersons, manufacturers, and office workers than that in men with technical jobs after adjustment for covariates in Japanese men. In conclusion, occupation type was an independent factor for the development of type 2 diabetes in Japanese men. The findings of this study may be important for the advancement of healthcare in diabetes.

## Data availability statement

The raw data supporting the conclusions of this article will be made available by the authors, without undue reservation.

## Ethics statement

The studies involving human participants were reviewed and approved by the local Ethics Committee of the Panasonic Health Insurance Organization (approval number: 2021-001). Written informed consent for participation was not required for this study in accordance with the national legislation and the institutional requirements.

## Author contributions

MHab collected the data and wrote the manuscript. MHam, KK, and HM contributed to the discussion. HO and MI analyzed the data and contributed to the discussion. MF reviewed and edited the manuscript. All authors contributed to the article and approved the submitted version.
